# Implantation of a nerve protector embedded with human GMSC-derived Schwann-like cells accelerates regeneration of crush-injured rat sciatic nerves

**DOI:** 10.1186/s13287-022-02947-4

**Published:** 2022-06-20

**Authors:** Qunzhou Zhang, Justin C. Burrell, Jincheng Zeng, Faizan I. Motiwala, Shihong Shi, D. Kacy Cullen, Anh D. Le

**Affiliations:** 1 Department of Oral and Maxillofacial Surgery and Pharmacology, University of Pennsylvania School of Dental Medicine, 240 South 40th Street, Philadelphia, PA 19104 USA; 2 Department of Neurosurgery, Center for Brain Injury and Repair, Perelman School of Medicine, University of Pennsylvania, Philadelphia, PA USA; 3 Department of Bioengineering, School of Engineering and Applied Science, University of Pennsylvania, Philadelphia, PA USA; 4 Center for Neurotrauma, Neurodegeneration and Restoration, Corporal Michael J. Crescenz Veterans Affairs Medical Center, Philadelphia, PA 19104 USA; 5 Guangdong Provincial Key Laboratory of Medical Molecular Diagnostics, Guangdong Key Laboratory of Medical Bioactive Molecular Developmental and Translational Research, Guangdong Medical University, Dongguan, 523808 China; 6 Department of Oral and Maxillofacial Surgery, Perelman Center for Advanced Medicine, Penn Medicine Hospital of the University of Pennsylvania, 3400 Civic Center Blvd, Philadelphia, PA 19104 USA

**Keywords:** Gingiva-derived mesenchymal stem cells, Collagen hydrogel, Schwann-like cells, Macrophages, Nerve regeneration

## Abstract

**Background:**

Peripheral nerve injuries (PNIs) remain one of the great clinical challenges because of their considerable long-term disability potential. Postnatal neural crest-derived multipotent stem cells, including gingiva-derived mesenchymal stem cells (GMSCs), represent a promising source of seed cells for tissue engineering and regenerative therapy of various disorders, including PNIs. Here, we generated GMSC-repopulated nerve protectors and evaluated their therapeutic effects in a crush injury model of rat sciatic nerves.

**Methods:**

GMSCs were mixed in methacrylated collagen and cultured for 48 h, allowing the conversion of GMSCs into Schwann-like cells (GiSCs). The phenotype of GiSCs was verified by fluorescence studies on the expression of Schwann cell markers. GMSCs encapsulated in the methacrylated 3D-collagen hydrogel were co-cultured with THP-1-derived macrophages, and the secretion of anti-inflammatory cytokine IL-10 or inflammatory cytokines TNF-α and IL-1β in the supernatant was determined by ELISA. In addition, GMSCs mixed in the methacrylated collagen were filled into a nerve protector made from the decellularized small intestine submucosal extracellular matrix (SIS-ECM) and cultured for 24 h, allowing the generation of functionalized nerve protectors repopulated with GiSCs. We implanted the nerve protector to wrap the injury site of rat sciatic nerves and performed functional and histological assessments 4 weeks post-surgery.

**Results:**

GMSCs encapsulated in the methacrylated 3D-collagen hydrogel were directly converted into Schwann-like cells (GiSCs) characterized by the expression of S-100β, p75NTR, BDNF, and GDNF. In vitro, co-culture of GMSCs encapsulated in the 3D-collagen hydrogel with macrophages remarkably increased the secretion of IL-10, an anti-inflammatory cytokine characteristic of pro-regenerative (M2) macrophages, but robustly reduced LPS-stimulated secretion of TNF-1α and IL-1β, two cytokines characteristic of pro-inflammatory (M1) macrophages. In addition, our results indicate that implantation of functionalized nerve protectors repopulated with GiSCs significantly accelerated functional recovery and axonal regeneration of crush-injured rat sciatic nerves accompanied by increased infiltration of pro-regenerative (M2) macrophages while a decreased infiltration of pro-inflammatory (M1) macrophages.

**Conclusions:**

Collectively, these findings suggest that Schwann-like cells converted from GMSCs represent a promising source of supportive cells for regenerative therapy of PNI through their dual functions, neurotrophic effects, and immunomodulation of pro-inflammatory (M1)/pro-regenerative (M2) macrophages.

**Supplementary Information:**

The online version contains supplementary material available at 10.1186/s13287-022-02947-4.

## Background

Peripheral nerve injuries (PNIs) represent one of the major challenges in the clinic due to the considerable long-term disability potential [1–3]. Upon injury, the injured nerves quickly undergo Wallerian degeneration characterized by dedifferentiation of mature Schwann cells into a repair phenotype with increased phagocytic, migratory, and secretory functions, followed by the recruitment of macrophages to aid the clearance of axonal and myelin debris [2, 4, 5]. Therefore, the interaction and cooperation between Schwann cells and macrophages play a critical role in the whole process of peripheral nerve regeneration after injury [6, 7]. Since peripheral nerves possess the limited intrinsic regenerative capability, the outcome for functional recovery is usually poor, especially when the injury is severe and/or a timely intervention is not achievable [5, 8, 9]. To date, nerve autografts remain the gold standard for the repair of severe PNIs, but several major shortcomings, e.g., a limited source of donor nerve tissues, the need for a secondary surgical procedure and related donor site morbidities, and the mismatch in nerve type and size, significantly restricted their clinical application [9, 10]. Clearly, there is an unmet clinical need for the development of new devices such as different types of nerve guidance conduits or nerve protectors as alternative approaches to facilitate regeneration and functional recovery of severe PNIs [11].

In recent years, much progress has been made in fabricating functionalized nerve guidance conduits loaded with different types of bioactive noncellular or cellular components, particularly the combination with multipotent mesenchymal stem cells (MSCs) of different tissue origins, including dental tissues [12, 13]. Accumulating evidence has shown that MSC-based tissue engineering and regenerative therapy holds great promise to promote nerve regeneration because of the potent immunomodulatory/anti-inflammatory functions and multifaced trophic effects of MSCs, such as neurotrophic or neuroprotective, pro-angiogenic, antioxidant, and anti-fibrotic effects due to their paracrine secretion of a myriad of biological factors [13, 14]. Interestingly, a unique population of MSCs has been identified and isolated from several neural crest (NC)-derived postnatal tissues, such as dental pulp [15], oral mucosa/gingiva [16–18], and skin epidermis [19]. Under special in vitro culture conditions, for example crestosphere culture, these NC-derived cells tend to maintain their neural crest stem-like cell (NCSC) properties [15–17, 20]. Adult NCSCs may serve as an important source of seed cells for developing novel tissue engineering and regenerative medicine (TE/RM) products, particularly those to support nerve regeneration due to their intrinsic ability to differentiate into glial or Schwann-like cells [21, 22]. However, one of the challenges for the use of adult NCSCs or their derivative Schwann-like cells in TE/RM is how to acquire a sufficient number of them without losing their NCSC properties during the progressive expansion process in vitro [22]. Recently, several studies have demonstrated the direct conversion of somatic cells, such as fibroblasts and epidermal keratinocytes, into NCSC-like cells [23–27] or Schwann-like cells [28–30] through the genetic introduction of certain transcriptional factors or some non-genetic approaches.

Gingiva-derived mesenchymal stem cells (GMSCs) possess multipotent differentiation capabilities and potent immunomodulatory/anti-inflammatory functions and have shown therapeutic potentials in various animal models of human diseases [31, 32]. MSCs derived from different tissues, e.g., bone marrow, adipose, and umbilical cord, etc., can be induced into Schwann-like cells under special induction conditions [33]. Our recent study showed that GMSCs could directly convert into NCSC-like cells under defined culture conditions [34] possibly due to their neural crest origin [18, 35, 36]. Most recently, we demonstrated the feasibility to generate functionalized neural guidance conduits through harnessing 3D collagen hydrogel-directed rapid conversion of GMSCs into Schwann-like cells (GiSCs), which displayed significant therapeutic potentials to facilitate the functional recovery and axonal regeneration of transected rat facial nerves [37]. In the present study, we aim to further explore the immunomodulatory effect of GiSCs encapsulated in 3D-collagen hydrogel on macrophages and their therapeutic effects in a sciatic nerve crush injury model in rats. Our results showed that GMSCs encapsulated in the methacrylated 3D-collagen hydrogel directly converted into Schwann-like cells characterized by increased expression of Schwann cell markers and neurotrophic factors. Meanwhile, we showed that these GiSCs encapsulated in the 3D-collagen hydrogel retained potent immunosuppressive effects on the activation of pro-inflammatory (M1) macrophages and, concomitantly, promoted the polarization of pro-regenerative (M2) macrophage polarization. In vivo, we found that implantation of functionalized nerve protectors (NPs) laden with GiSCs significantly improved functional recovery and axonal regeneration of the injured nerves accompanied by increased infiltration of pro-regenerative (M2) macrophages and reduced infiltration of pro-inflammatory (M1) macrophages. These findings support the notion that GiSCs promote nerve regeneration possibly due to their neurotrophic effects and immunomodulatory functions on the phenotype and activation of macrophages.

## Methods

### Animals

Female Sprague–Dawley rats aged 6–8 weeks old (weighing 200–250 g) were purchased from Charles River Laboratories and housed in controlled animal facilities with a temperature of 23 °C ± 2 °C, a humidity of 40–65%, and a 12/12-h light/dark cycle. Animals were fed a standard laboratory diet and allowed ad libitum access to drinking water. All animal procedures were approved by the Institutional Animal Care and Use Committee (IACUC) of the University of Pennsylvania.

### Cell cultures

Gingival tissues were obtained as discarded tissues from healthy human subjects (aged from 20 to 40 years) who underwent a dental procedure, which was approved by the Institutional Review Board (IRB) at the University of Pennsylvania, while informed consent forms were obtained from the subjects. Primary GMSCs were routinely isolated, characterized, and maintained in our laboratory as described previously [32, 37]. Briefly, GMSCs were cultured in the complete culture medium: α-minimum essential medium (α-MEM: Invitrogen) supplemented with 10% fetal bovine serum (FBS: Zen-Bio, Inc., Durham, NC), 1% antibiotics (100U/ml penicillin/100 µg/ml streptomycin; Invitrogen), 2 mM L-glutamine, 100 mM non-essential amino acid (NEAA), and 550 μM 2-mercaptoethanol (2-ME; Sigma-Aldrich) and cultured at 37 °C in a humidified tissue culture incubator with 5% CO_2_. The adherent confluent cells were passaged with 0.05% trypsin containing 1 mM EDTA and continuously sub-cultured in the complete growth medium. Cells less than sixth passages were used in the experiments [32].

THP-1 cells, a human acute monocytic leukemic cell line, were obtained from ATCC (TIB-202) and cultured in an incubator at 37 °C, 5% CO_2_, and 95% humidity in RPMI-1640 medium containing 2 mM L-glutamine, 25 mM HEPES, 1% sodium pyruvate, 0.01% of 2-mercaptoethanol (2-ME), 10% fetal bovine serum (FBS), and 1% penicillin/streptomycin. All cell culture media and supplements were purchased from Invitrogen (CA, USA).

### Preparation of the 3D-collagen hydrogel encapsulated with GMSCs

A purified methacrylated Type I bovine collagen (> 98%) was purchased from Advanced BioMatrix, Inc. (Carlsbad, CA). A stock solution of collagen hydrogel at a 6 mg/mL concentration was prepared according to the manufacturer’s instructions and our recent studies [37]. Briefly, a calculated volume of the chilled neutralization solution (NS) and collagen stock solution was mixed thoroughly by pipetting, followed by adding GMSCs resuspended in a calculated volume of chilled PBS into the mixture. According to our recent study, an optimized final concentration of the collagen hydrogel at 4 mg/mL and a cell density at 2 × 10^6^/mL were selected throughout our present study [37]. Then, the mixture was aliquoted into a Petri culture dish and incubated at 37 °C for 20 min allowing for hydrogel formation, followed by culturing for 48 h in the complete culture medium (α-MEM supplemented with 10% FBS, 1% antibiotics, 2 mM L-glutamine, 100 mM NEAA, and 550 μM 2-ME) in the absence of extra mitogens, e.g., basic fibroblast growth factor (bFGF) and platelet-derived growth factor AA (PDGF-AA), neurotrophic factors, e.g., heregulin-β-1, and chemicals, e.g., all trans-retinoic acid (RA) and forskolin, all of which are commonly used for the induction of Schwann-like cells from MSCs [33, 34]. The cell-laden 3D collagen constructs were harvested, and cryosections were prepared for further analysis [37].

### Immunofluorescence studies

Cryosections (10 µm thickness) prepared from the 3D-collagen hydrogel encapsulated with GMSCs were permeabilized in 0.5% Triton X‐100 for 20 min and blocked with 2.5% goat serum in PBS at room temperature for 1 h. Then, the sections were incubated with the following primary antibodies at 4 °C overnight: S-100β (M00979-1; rabbit monoclonal IgG, 1:200; Boster, Pleasanton, CA), p75 (AHP1014; rabbit IgG, 1:200; BioRad), BDNF (ab108319; rabbit IgG, 1:200; Abcam), and GDNF (ab18956; rabbit IgG, 1:200; Abcam). Following washing twice with PBS, sections were incubated with Alexa Fluor^®^ 488 Donkey anti-rabbit IgG (minimal x-reactivity) antibody (406,416; 1:300, BioLegend) at room temperature for 1 h, while an isotype-matched control antibody, FITC Donkey anti-rabbit IgG (minimal x-reactivity) antibody (BioLegend), was used as a negative control. Nuclei were counterstained with 4’,6-diamidino-2-phenylindole (DAPI). Images were captured using Olympus inverted fluorescence microscope (IX73). For semiquantitative analysis, cells with positive signals in at least six random high-power fields (HPF) were visualized, counted, and expressed as the percentage of total DAPI-positive cells [32, 37].

### Co-culture of GMSCs and THP-1 macrophages

THP-1 cells were seeded into a 6-well culture plate (1 × 10^6^/well), followed by treatment with 100 nM phorbol 12-myristate 13-acetate (PMA; Sigma) in RPMI-1640 culture media for 6 h to induce differentiation of THP-1 cells into M0 macrophages [38]. Then, the media was removed and cells were washed twice with PBS. Following resting for 24 h in serum-free RPMI-1640, differentiated THP-1 macrophages were indirectly co-cultured with 5 × 10^5^ of GMSCs at a cell ratio of 1:2 (GMSC/THP-1 cells) that were seeded onto the top cell insert with 1-µm-sized pores (Fisher Scientific) [39]. Otherwise, GMSCs (5 × 10^5^) encapsulated in the 3D-collagen hydrogel (4 mg/mL) at a final cell density of 2 × 10^6^/mL were directly placed into a 6-well culture plate containing differentiated THP-1 macrophages (1 × 10^6^/well). Cells were continuously cultured in complete RPMI-1640 culture media for 48 h, followed by stimulation with 100 ng/mL of lipopolysaccharide (LPS) for 3 h. Then, the conditioned culture media were harvested for ELISA on the secretion of IL-10, IL-1β, and TNF-α. Under certain conditions, THP-1 cells were co-cultured with GMSCs for 24 h followed by stimulation with 100 ng/mL of LPS for 24 h to induce M1 macrophages. For all experiments, GMSCs and THP-1 macrophages cultured alone served as controls.

### Enzyme-linked immunosorbent assay (ELISA)

The secretion level of IL-10, IL-1β, and TNF-*α* in the supernatants of co-cultured cells was detected using the ELISA MAX™ Deluxe Sets according to the manufacturer’s protocols (BioLegend; San Diego, CA).

### Generation of a functionalized nerve protector laden with GMSC-derived Schwann-like cells

Functionalized nerve protectors were generated according to the established procedures as described previously [37]. Briefly, about 40 µl of methacrylated collagen hydrogel (4 mg/mL) encapsulated with GMSCs (2 × 10^6^/mL) was filled into customized nerve protectors (NPs) (2 mm in internal diameter × 10 mm length) made of porcine small intestine submucosal extracellular matrix (SIS-ECM) (Cook Biotech, West Lafayette, IN) and incubated at 37 °C for 20 min, followed by continuously culturing in complete α-MEM medium for 24 h.

### Crush injury of rat sciatic nerves and implantation of functionalized nerve protector

Rats were anesthetized by intraperitoneal injection of a mixture of ketamine/xylazine (100/10 mg/kg body weight). An incision was made from the right sciatic notch to the distal thigh, and the subcutaneous tissue was bluntly dissected to expose the bicep femoris muscle. The sciatic nerve was exposed and crushed at a point 5 mm distal to the sciatic notch with a type 5 watchmaker forceps for 30 s as previously described [40, 41]. Then, the empty or functionalized NPs (10 mm in length) laden with GiSCs were wrapped around the injury site, while rats with crush injuries alone served as the control. Four weeks following nerve injury and implantation of nerve protectors, the animals were killed and the sciatic nerves were harvested for further analysis.

### Rat sciatic functional index (SFI) analysis

At 4 weeks post-injury and implantation of nerve conduits, rats with hind paws dipped in black ink were guided to walk across a narrow track, and footprints were recorded on white paper. Afterward, the following parameters on both the normal (N) and the experimental (E) hind legs were measured: print length (PL), the distance from the heel to the toe; toe spread (TS), the distance from the first to the fifth toes; and intermediary toe spread (ITS), the distance from the second to the fourth toes. SFI was calculated according to the following formula: SFI =  − 38.3 × (EPL − NPL)/NPL + 109.5 × (ETS − NTS)/NTS + 13.3 × (EITS − NITS)/NITS − 8.8.

The SFI varies from 0 to − 100: scores at about 0 represent a normal nerve function, while scores at about − 100 represent a complete loss of function [40].

### Electrophysiological analysis

Electrophysiological analysis was performed at 4 weeks post-crush injury of sciatic nerves of rats [40]. Bipolar stimulating electrodes were placed percutaneously either proximal or distal to the sciatic nerve injury, and a subdermal recording electrode was placed in the tibialis anterior muscle with a reference electrode placed in the tendon. After determining the initial threshold for an evoked muscle response, the supramaximal compound muscle action potential (CMAP) was obtained by doubling the current until the waveform plateaued and then averaged over a train of 5 pulses (0–5 mV; 100 × gain; 10–10,000 Hz bandpass and 60 Hz notch filters; Natus Viking EDX). CMAP amplitude and the time latency following proximal or distal stimulation were measured. Motor nerve conduction velocity (NCV) was calculated based on the difference in latency and distance between the two different stimulation points across the crush injury site of the sciatic nerve. CMAP latency was estimated as the time between the stimulus artifact and the first depolarization at the start of the CMAP. CMAP percent recovery was calculated by normalizing the ipsilateral response to the contralateral, uninjured side.

### Immunohistochemical studies

The gastrocnemius muscles of both hindlimbs were harvested and weighed at 4 weeks post-injury and implantation of nerve protectors. The dissected nerve tissue samples were fixed in 4% PFA for 24 h and cryoprotected in 10%, 20%, and 30% sucrose and embedded in O.C.T., and 10-µm-thick cryosections were cut. After permeabilization in 0.5%Triton X‐100 for 20 min and blocking with 2.5% goat serum in PBS at room temperature for 1 h, the sections were incubated with primary antibodies at 4 °C overnight: S-100β (M00979-1; rabbit monoclonal IgG, 1:200; Boster), β-tubulin III (MCA2047; mIgG1, 1:200; BioRad), human nuclei (GTX82624; mIgG, 1:200; GenTex), BDNF (ab108319; rabbit IgG, 1:200; Abcam), GDNF (ab18956; rabbit IgG, 1:200; Abcam), active caspase-3 (AB3623; rabbit IgG, 1:200; Millipore), CD68 (MCA341GA; mouse IgG, 1:200; BioRad), arginase-1 (16,001–1-AP; rabbit IgG, 1:200; Proteintech), iNOS (18,985–1-AP; rabbit IgG, 1:200; Proteintech), and CD206 (18,704–1-AP; rabbit IgG, 1:200; Proteintech). After washing twice with PBS, the sections were incubated at room temperature for 1 h with Alexa Fluor^®^ 488 Donkey anti-rabbit IgG (minimal x-reactivity) antibody (406,416; 1:300, BioLegend) and Alexa Fluor^®^ 588 goat anti-mouse IgG (minimal x-reactivity) antibody (405,326; 1:300, BioLegend), while corresponding isotype-matched control antibodies (BioLegend) were used as negative controls. Nuclei were counterstained with 4’,6-diamidino-2-phenylindole (DAPI). Images were captured using an Olympus inverted fluorescence microscope (IX73), and the integrated immunofluorescence intensity for each protein expression in six randomly selected regions of interest (ROI) was quantified using the Olympus cellSens Dimension software [37]. To quantify the expression of S-100β, GDNF, and BDNF in transplanted human GMSCs, the area of colocalized immunolabeling signals (in yellow-orange pixels) of these proteins (in green color) and human nuclei (in red color) in the merged files was measured using ImageJ program and presented as the percentage of colocalization = the area of yellow-orange pixels/total area of green pixels [42]. To quantify the apoptosis of transplanted human MSCs, the area of colocalized immunolabeling signals (in yellow-orange pixels) of active caspase-3 (in green color) and human nuclei (in red color) in the merged files was measured using ImageJ program and presented as the percentage of colocalization = the area of yellow-orange pixels/total area of red pixels [42].

### Morphological evaluation of rat sciatic nerves

The dissected sciatic nerves were fixed with 2.5% glutaraldehyde overnight at 4 °C and postfixed with 1% osmium tetroxide (OsO4) for 2 h, dehydrated, and embedded in epoxy resin. Semi-thin sections (1 µm) were cut vertically with an ultramicrotome (EM UC7i, Leica Microsystems, Denver, CO, http://www.leica-microsystems.com) and stained with 1% toluidine blue solution, and images were captured under a light microscope (Olympus IX-73). The density of the myelinated fibers (fibers/1000 µm^2^) was analyzed from six non-overlapping visual fields per specimen. On the other hand, ultrathin sections (60 nm) were stained with lead citrate and uranyl acetate, and images were captured under a transmission electron microscope (TEM, JEM-1400). All these services were provided by the Electron Microscopy Resource Lab of Perelman School of Medicine at UPenn. The diameter of myelinated fibers, axons, and the thickness of the myelin sheath was evaluated by cellSens Dimension software (Olympus), and the G-ratio was calculated as the ratio of the inner axonal diameter to the total outer diameter of the fiber.

### Statistical analysis

All data were expressed as mean ± standard error of measurement (SEM), and all statistical analyses were carried out using *SPSS Statistics version 18.0* (IBM, Inc., Armonk, NY, USA). Direct comparisons between experimental and control groups were analyzed by paired Student’s *t test*. One-way analysis of variance (ANOVA) was employed for multiple comparisons. Post hoc pairwise comparison between individual groups was performed using Tukey’s test. A *P* value of less than 0.05 was considered statistically significant.

## Results

### GMSCs encapsulated in the 3D-collagen hydrogel converted into Schwann-like cells

**Fig. 1:**
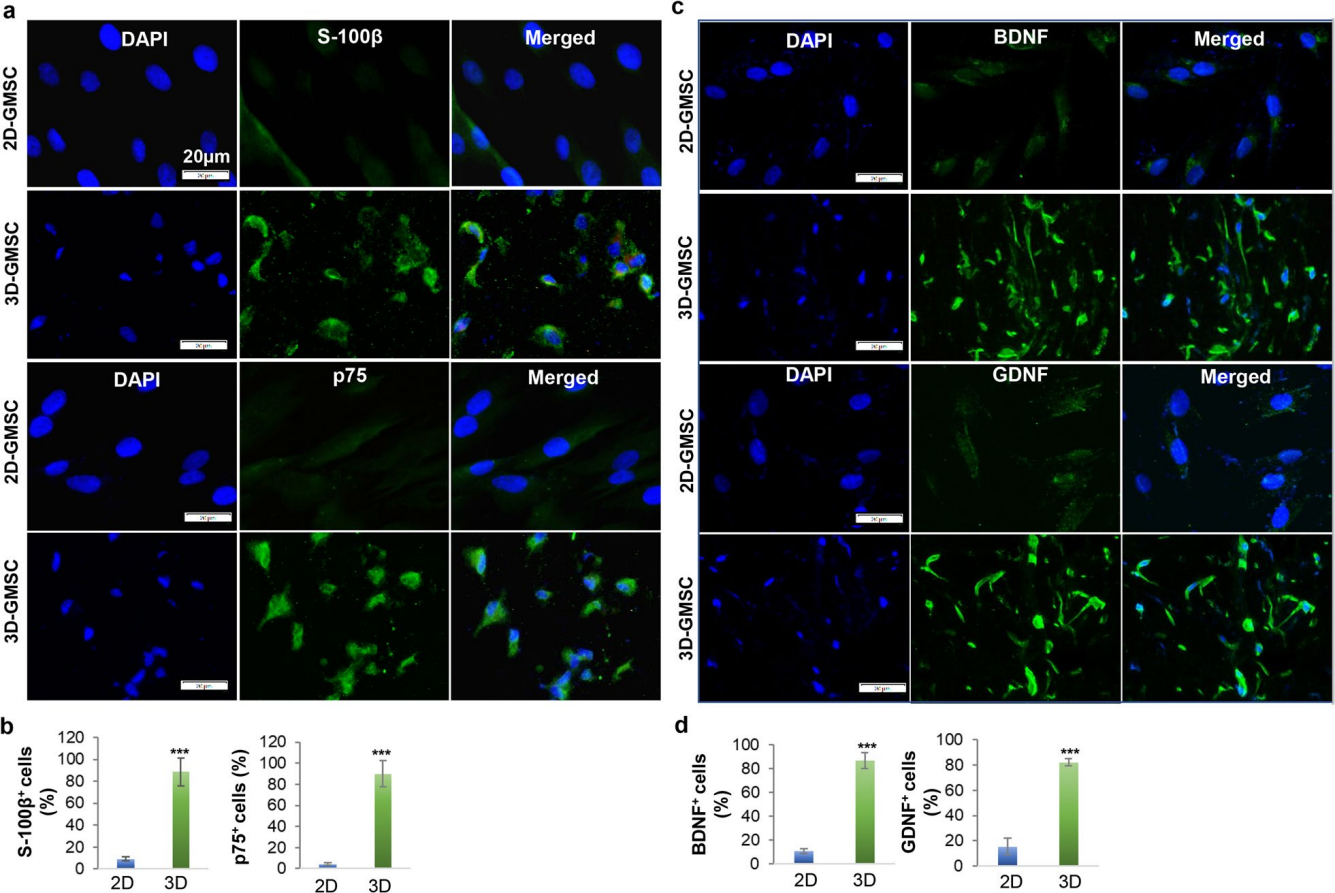
3D-collagen hydrogel directed the conversion of GMSCs into Schwann-like cells. GMSCs were cultured under 2D-culture conditions (2D-GMSC) or encapsulated in the methacrylated 3D-collagen hydrogel (4 mg/mL) at a cell density of 2 × 10^6^/mL and allowed for gel formation at 37 °C for 20 min followed by culturing in complete α-MEM supplemented with 10% FBS for 48 h. **a** 2D- or 3D-GMSCs were immunostained with a specific antibody for S-100β or p75NTR followed by incubation with Alexa Fluor 488-conjugated secondary antibodies. Nuclei were counterstained with 4’,6-diamidino-2-phenylindole (DAPI; blue). Images were captured under a fluorescence microscope. Scale bars, 20 µm. **b** Quantification of the percentage of S-100β^+^ and p75NTR^+^ cells. **c** 2D- or 3D-GMSCs were immunostained with a specific antibody for BDNF or GDNF followed by incubation with Alexa Fluor 488-conjugated secondary antibodies. Nuclei were counterstained with DAPI (blue). Images were captured under a fluorescence microscope. Scale bars, 20 µm. **d** Quantification of the percentage of BDNF^+^ and GDNF^+^ cells. Data represent the mean ± SD. ***p < 0.001 (3D *vs.* 2D); Student’s two-tailed unpaired *t* test (B, D). 2D, GMSCs cultured in 2D-conditions; 3D, GMSC cultured in the 3D-collagen hydrogel

Most recently, we demonstrated that the methacrylated 3D-collagen hydrogel with an optimal stiffness drives the direct conversion of GMSCs into Schwann cell precursor-like cells (designated as GiSCs) [37]. Herein, immunofluorescence studies further showed that GMSCs displayed a significant increase in the protein expression of S-100β and p75^NTR^, two common markers for Schwann cell precursors [43] when they were encapsulated and cultured in the methacrylated 3D-collagen hydrogel at a concentration of 4 mg/mL for 48 h compared to their counterparts under 2D-culture conditions (Fig. 1a, b). Meanwhile, 3D-cultured GMSCs also had a remarkably increased expression of neurotrophic factors, brain-derived neurotrophic factor (BDNF), and glial cell-derived neurotrophic factor (GDNF), compared with 2D-cultured counterparts (Fig. 1c, d). These findings further support that GMSCs encapsulated in the 3D-collagen hydrogel can be directly converted into Schwann-like cells with increased expression of neurotrophic factors.

### GMSCs encapsulated in the 3D-collagen hydrogel retained their immunomodulatory effects on macrophages

**Fig. 2:**
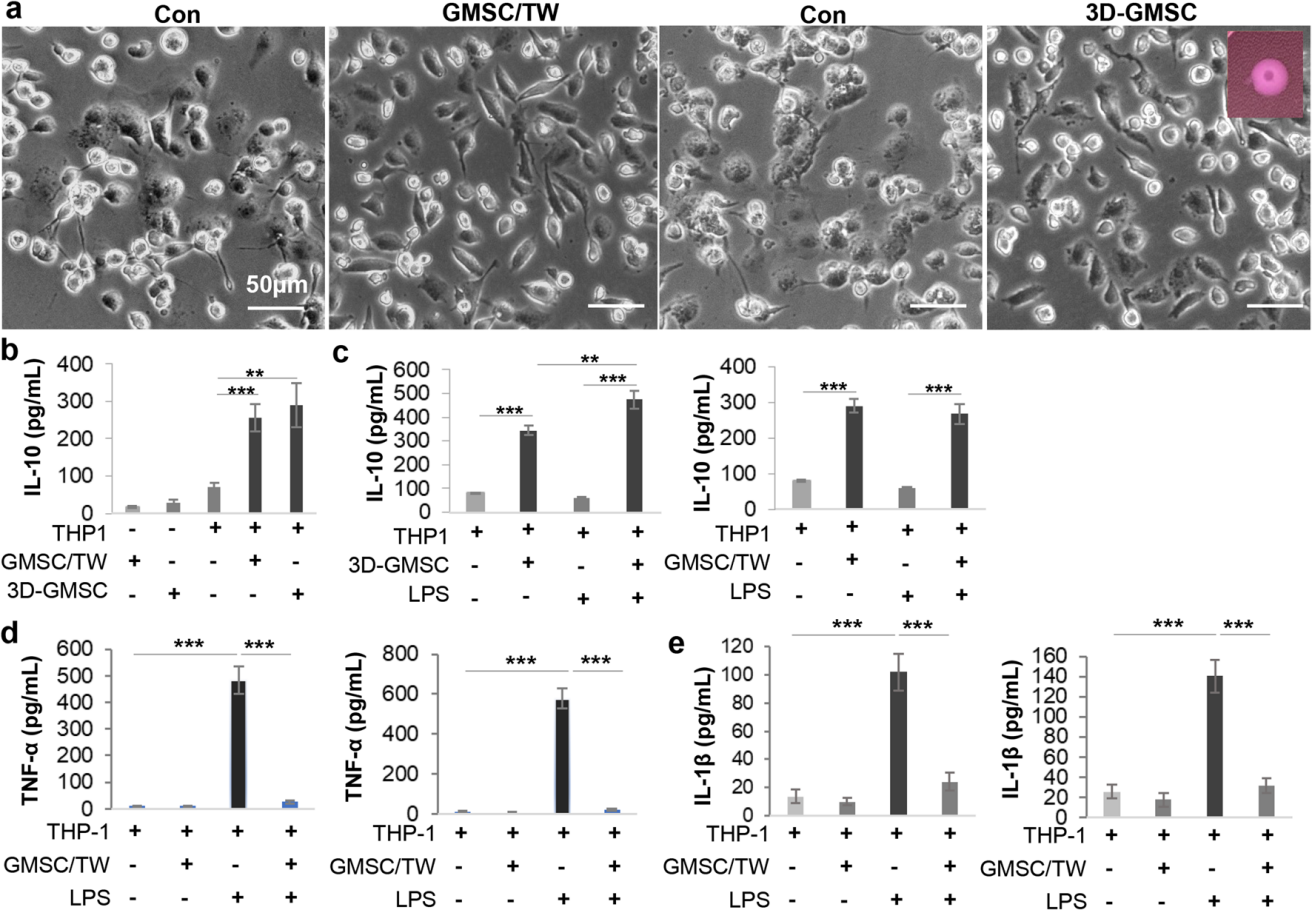
GMSCs encapsulated in the 3D-collagen hydrogel retain the immunomodulatory effects on macrophages. THP-1 macrophages were co-cultured with GMSCs either seeded in the upper chamber of a trans-well (TW) or encapsulated in the methacrylated 3D-collagen hydrogel (4 mg/mL) at a cell ratio of 2:1 (macrophages: GMSCs) for 48 h. **a** The spindle-shaped morphological changes of THP-1 macrophages following co-culture with GMSCs. Scale bar, 50 µm. **b** Following co-culture with GMSCs for 48 h, the secretion of IL-10 in the culture media was determined by ELISA. **c-e** Following co-culture with GMSCs for 48 h, THP-1 macrophages were stimulated with 100 ng/mL lipopolysaccharide (LPS) in fresh media for 3 h and the secretion of IL-10 (**c**), TNF-α (**d**), and IL-1β (**e**) was determined by ELISA, respectively. Data represent the mean ± SD, n = 3 biological replicates. **p < 0.01; ***p < 0.001; Student’s two-tailed unpaired t test. TW, GMSCs cultured in a trans-well; 3D-GMSC, GMSC cultured in the 3D-collagen hydrogel

Macrophages play a critical cooperative role with Schwann cells in nerve regeneration after injury [6, 7]. Our previous study showed that GMSCs can potently promote the polarization of pro-regenerative (M2) macrophages while suppressing the activation of pro-inflammatory (M1) macrophages [39]. We then asked whether Schwann-like cells converted from GMSCs encapsulated in the 3D-collagen hydrogel retained their immunomodulatory effects on macrophages. For this purpose, the methacrylated 3D-collagen hydrogel encapsulated with 5 × 10^5^ of GMSCs (at a cell density of 2 × 10^6^) was directly placed into a 6-well culture plate seeded with THP-1 derived M0 macrophages (1 × 10^6^/well) (Fig. 2a). Otherwise, the same number of GMSCs was indirectly co-cultured with 1 × 10^6^ of THP-1 derived M0 macrophages (1:2) in a trans-well system as previously described [39]. Following co-culture for 48 h, with GMSCs either in the trans-well or encapsulated in the 3D-collagen hydrogel, THP-1 macrophages displayed elongated cellular morphology characteristic of a pro-regenerative M2-like phenotype [44, 45]. Concomitantly, co-culture with GMSCs under two conditions led to a comparable increase in the secretion of IL-10, a signature anti-inflammatory cytokine of pro-regenerative (M2) macrophages, compared with THP-1 macrophages or GMSCs cultured alone (Fig. 2b). Under certain conditions, THP-1 M0 macrophages were co-cultured with GMSCs under two conditions for 48 h and then stimulated with 100 ng/mL of LPS in fresh culture media for 3 h. Our results indicated that co-culture with GMSCs in the trans-well or with 3D-GMSCs not only increased IL-10 secretion (Fig. 2c) but also significantly reduced the secretion of TNF-α and IL-1β (Fig. 2d, e), two common pro-inflammatory cytokines secreted by anti-inflammatory (M1) macrophages. These results suggest that Schwann-like cells converted from GMSCs encapsulated in the 3D-collagen hydrogel retained their potent capability to promote polarization of pro-regenerative (M2) macrophages and suppress the activation of pro-inflammatory (M1) macrophages.

### *The fate of GMSC-derived Schwann-like cells following transplantation *in vivo

Most recently, we demonstrated the feasibility to generate functionalized neural guidance conduits by harnessing the 3D collagen hydrogel-directed conversion of GMSCs into Schwann-like cells (GiSCs) [37]. Using the same approach, we herein confirmed the successful fabrication of functionalized nerve protectors (NPs) made of porcine small intestine submucosal (SIS) extracellular matrix, whereby the decellularized wall matrix of NPs were repopulated with GiSCs as evidenced by the positive expression of S-100β in cells that have transmigrated into the wall matrix (data not shown).

**Fig. 3:**
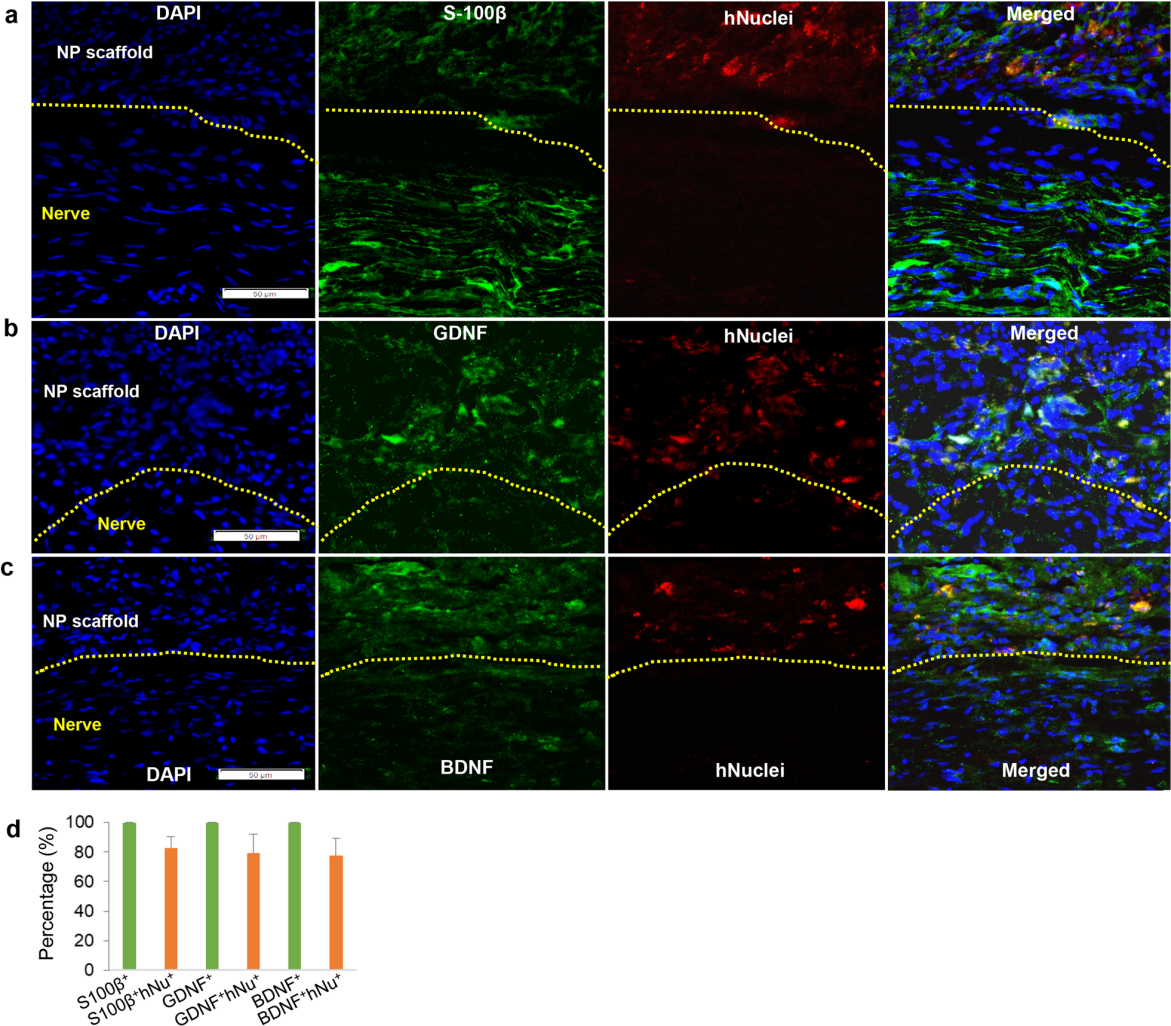
The fate of GMSC-derived Schwann-like cells following transplantation within the functionalized nerve proctor to the crush injury site of rat sciatic nerves. The functionalized nerve protectors repopulated with GMSC-derived Schwann-like cells were implanted to wrap the injury segment of rat sciatic nerves. Four weeks post-implantation, the injured nerves were harvested and cryosections were prepared for immunofluorescence studies. The longitudinal cryosections were incubated with a specific mouse monoclonal antibody for human nuclei (red color) in combination with a rabbit polyclonal antibody for S-100β (**a**), GDNF (**b**), or BDNF (**c**) followed by incubation with Alexa Fluor 488- and 594-conjugated secondary antibodies. Nuclei were counterstained with 4’,6-diamidino-2-phenylindole (DAPI; blue). **d** Quantification of the percentage of S-100β^+^, GDNF^+^, and BDNF^+^ cells (green color) co-immunostained with human nuclei (red color) by using ImageJ, which were designated as S-100β^+^hNu^+^, GDNF^+^hNu^+^, and BDNF^+^hNu^+^, respectively, whereby the percentage of total cells stained with green color was arbitrarily set as 100%. Images were captured under a fluorescence microscope. Scale bars, 50 µm. The dashed lines separated the longitudinally sectioned nerve tissues (the lower side) and implanted nerve protector (NP) scaffolds (the upper side)

Next, we observed the in vivo fate and behavior of GiSCs repopulating the wall matrix of NPs following implantation to wrap the crush injury site of rat sciatic nerves (Additional file 1: Fig.S1a). Four weeks post-implantation, the NPs were not absorbed and then harvested together with the nerves for further analysis (Additional file 1: Fig. S1a). This is in consistent with previous studies, whereby SIS nerve guidance conduits (NGCs) stably maintained their shape without collapsing for up to 8 weeks [46] and showed minimal-to-mild resorption by up to 12 weeks following implantation in vivo [47]. Immunofluorescence (IF) studies indicated that those transplanted GMSCs integrated into the wall matrix of NPs and localized in the peripheral areas outside of the injured nerves as recognized by the positive expression of human nuclei, whereas only about 5% of them were positively stained for the active form of caspase-3 (Additional file 1: Fig. S1b, c), a specific marker for apoptotic cells. In addition, we noticed that in the neural protector scaffold occupied area, about 80% of those infiltered cells positively expressing the Schwann cell marker S-100β (Fig. 3a), and neurotrophic factors, GDNF and BDNF (Fig. 3b, c), were co-immunostained with human nuclei (designated as S-100β^+^huNu^+^, GDNF^+^hNu^+^, and BDNF^+^hNu^+^ cells, respectively) (Fig. 3d). Taken together, these findings have demonstrated the high survivability and secretion of neurotrophic factors of GiSCs integrated into the wall matrix of NPs following transplantation into the nerve injury site.

### Implantation of functionalized nerve protectors laden with GMSC-derived Schwann-like cells facilitated functional recovery and axonal regeneration of crush-injured rat sciatic nerves

**Fig. 4:**
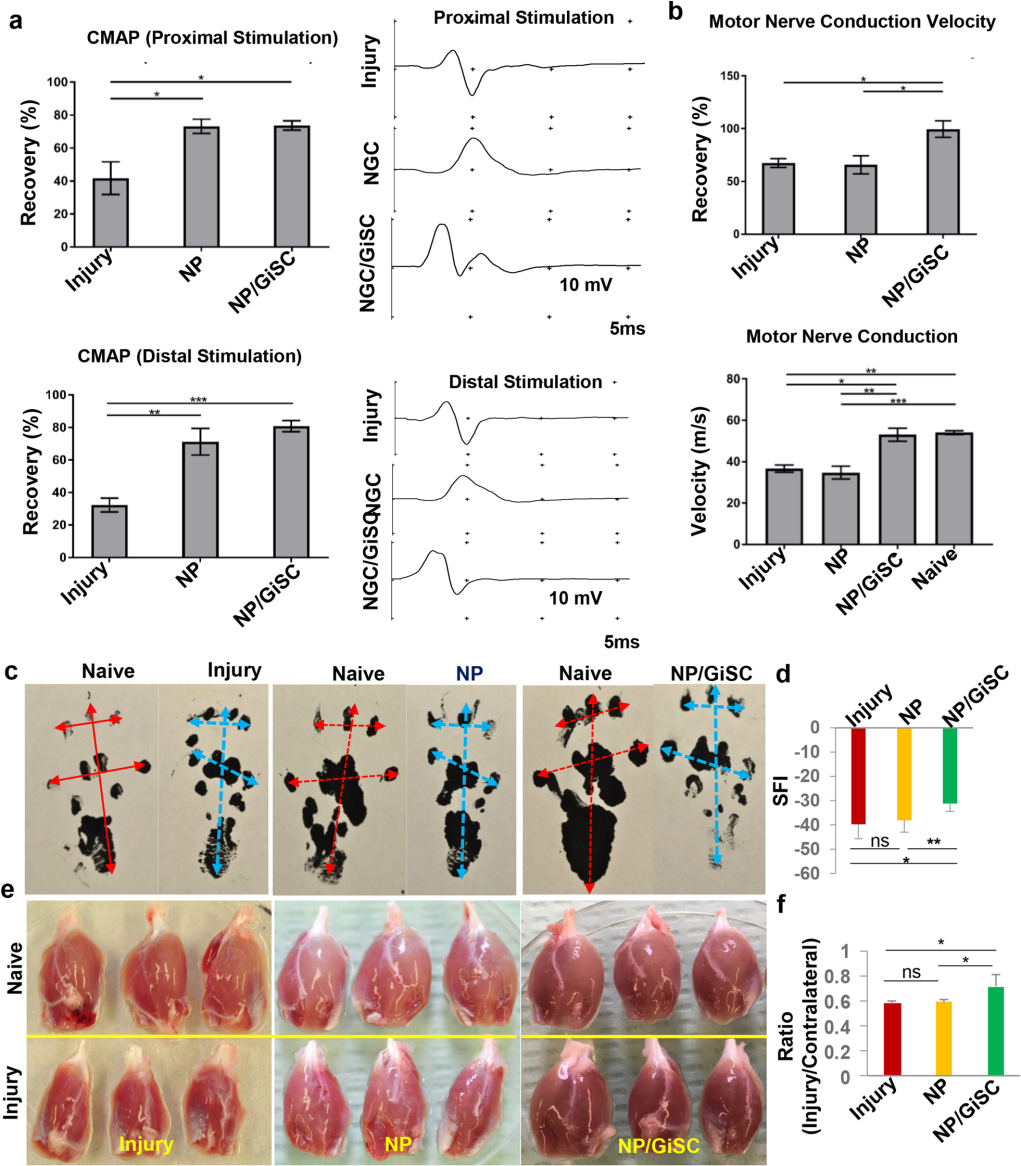
Implantation of nerve protectors repopulated with GMSC-derived Schwann-like cells improves functional recovery of crush-injured rat sciatic nerves. At 4 weeks post-injury and implantation, functional recovery of crush-injured sciatic nerves was analyzed. **a** Compound muscle action potential (CMAP) recordings of the gastrocnemius muscles of both the injury site and the contralateral naive side of rats (*n* = 4 for each group) following stimulation from either proximal or distal to the injury site. **b** Analysis of motor nerve conduction velocity of both the injury side and the contralateral normal side of rats (*n* = 4 for each group). **c**, **d** Measurement of foot printings and sciatic functional index (SFI). **e**, **f** Measurement of the wet weight of gastrocnemius muscles of all animals from different groups, and the ratio was calculated individually (ratio = the weight of the injury side/the weight of contralateral naive side; *n* = 6). Data are shown as the mean ± SD. *p < 0.05; **p < 0.01; ***p < 0.001; ns, no significance. One-way ANOVA with Tukey’s posttest. Abbreviations: NP, nerve protector; NP/GiSCs, nerve protector repopulated with GMSC-derived Schwann-like cells (GiSC)

We then determined the therapeutic potentials of the functionalized NP repopulated with GiSCs (NP/GiSC) following implantation to the crush-injured site of rat sciatic nerves (Additional file 1: Fig. S1a). At 4 weeks post-injury and implantation, EMG analysis indicated that implantation of NP/GiSCs and NP alone showed comparable beneficial effects on the recovery of compound muscle action potential (CMAP) with both proximal and distal stimulation (*p* < 0.05 *vs* injury control) (Fig. 4a). Interestingly, implantation of NP/GiSC showed much better effects on the recovery of motor nerve conduction velocity (*p* < 0.05, NP/GiSC *vs* NP) or the percentage of conduction velocity than NP alone (*p* < 0.01, NP/GiSC *vs* NP) (Fig. 4b). Consistently, walking track analysis showed that rats implanted with NP/GiSCs exhibited a significant improvement in the sciatic functional index (SFI) as compared to animals implanted with empty NP alone (*p* < 0.01, NP/GiSC *vs* NP) (Fig. 4c, d). In addition, we observed an overall loss of gastrocnemius muscle mass in all groups of animals at 4 weeks post-injury (Fig. 4e), and then the ratio of gastrocnemius muscle weight of the injured side to that of the contralateral side was calculated. The results showed that there was no significant difference in the average muscle ratios between the injury and empty NP groups (*p* > 0.05); however, the average muscle ratio of the NP/GiSC group was higher than that of either injury or empty NP groups (*p* < 0.05) (Fig. 4f), suggesting that implantation of NP/GiSC had better effects to prevent atrophy of gastrocnemius muscle than empty NP.

Histological examination of longitudinal sections of the injured sciatic nerves indicated that the nerve fibers at the injured sites in both empty NP and NP/GiSC implantation groups displayed a more organized and aligned axonal arrangement as compared with a random pattern of axonal growth presenting in the injury control group (Additional file 2: Fig. S2a). IF staining showed a decreased expression of S-100β and β-tubulin III in the injured nerve as compared to the intact normal nerve (*p* < 0.001) (Additional file 2: Fig. S2a–c). Implantation of empty NP or NP/GiSCs increased the expression of S-100β and β-tubulin III as compared with the injury control (*p* < 0.001, NP/GiSC *vs* injury; *p* < 0.01, empty NP *vs* injury), whereby implantation of NP/GiSC exhibited a more pronounced beneficial effect than empty NP (Additional file 2: Fig. S2a-c).

**Fig. 5:**
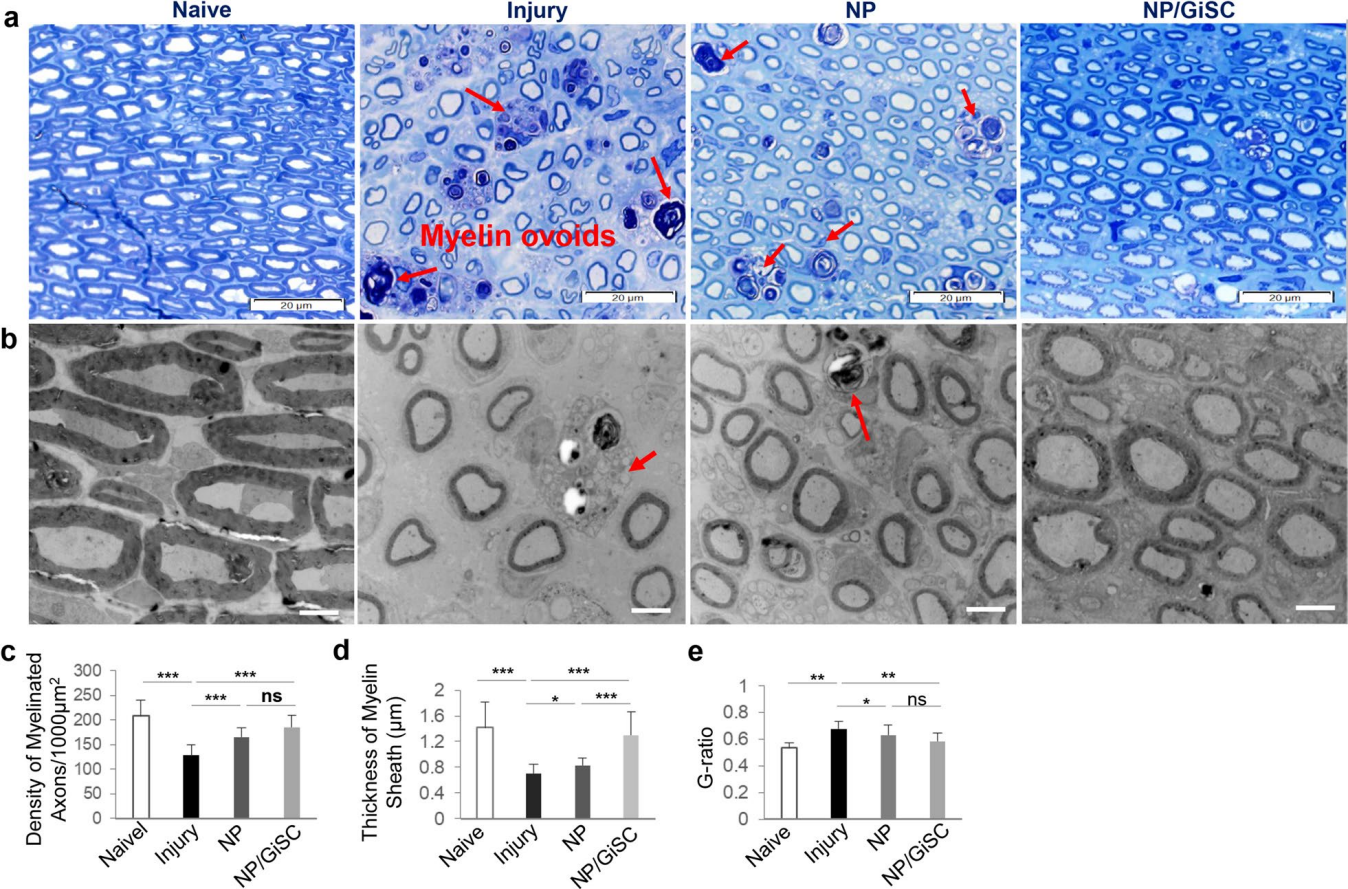
Implantation of nerve protectors repopulated with GMSC-derived Schwann-like cells promotes axonal regeneration and remyelination of crush-injured rat sciatic nerves. **a** Toluidine blue staining of semi-thin sections of the injured nerves from different groups of rats at 4 weeks post-injury and implantation. Scale bars, 20 µm. **b** Transmission electron microscopy (TEM) of ultrathin sections of the injured sciatic nerves from different groups of rats at 4 weeks post-injury and implantation. Scale bars, 4 µm. **c** Quantification of the density of myelinated axons (the number of myelinated axons/1000 µm^2^). **d** Quantification of the thickness of the myelin sheaths. **e** Calculation of the G-ratios (the inner axonal diameter/the outer myelinated fiber diameter). Data are shown as the mean ± SD. *p < 0.05, **p < 0.01, ***p < 0.001; ns, no significance. One-way ANOVA with Tukey’s posttest. Abbreviations: NP, nerve protector; NP/GiSCs, nerve protectors repopulated with GMSC-derived Schwann-like cells (GiSC)

Next, the remyelination of nerve fibers was evaluated by toluidine blue staining and transmission electron microscopy (TEM) (Fig. 5a, b). Morphologically, both toluidine blue staining and TEM showed that the control injured nerves revealed poorly regenerated nerves composed of thin, dispersed myelinated and non-myelinated nerve fibers in comparison with the normal control (Fig. 5a, b). As expected, crush injury led to a significant decrease in the density of myelinated nerve fibers and the average thickness of myelin sheath as compared to normal nerves (*p* < 0.001), but a relatively higher G-ratio (*p* < 0.01) as compared to the normal control (Fig. 5c-e). However, implantation of either empty NP or NP/GiSC significantly increased the density of myelinated nerve fibers as compared with the injury control (*p* < 0.001; NP or NP/GiSC *vs* injury), whereby NP/GiSC showed relatively better effects than empty NP at the border statistical significance (p = 0.064, NP/GiSC *vs* empty NP) (Fig. 5c). Further analysis showed that the myelin sheaths in empty NP and NP/GiSC groups were significantly thicker than those of the injury control (*p* < 0.05, empty NP *vs* injury; *p* < 0.001, NP/GiSC *vs* injury), while the myelin sheaths of NP/GiSC group were even thicker than those of empty NP group (*p* < 0.001, NP/GiSC *vs* empty NP) (Fig. 5d, e). Taken together, these findings demonstrated the regenerative therapeutic potentials of functionalized NPs laden with GiSCs (NP/GiSC) in the rat sciatic nerve crush injury model**.**

### Immunomodulatory effects of GMSC-derived Schwann-like cells on macrophages in rat sciatic nerves after crush injury

**Fig. 6:**
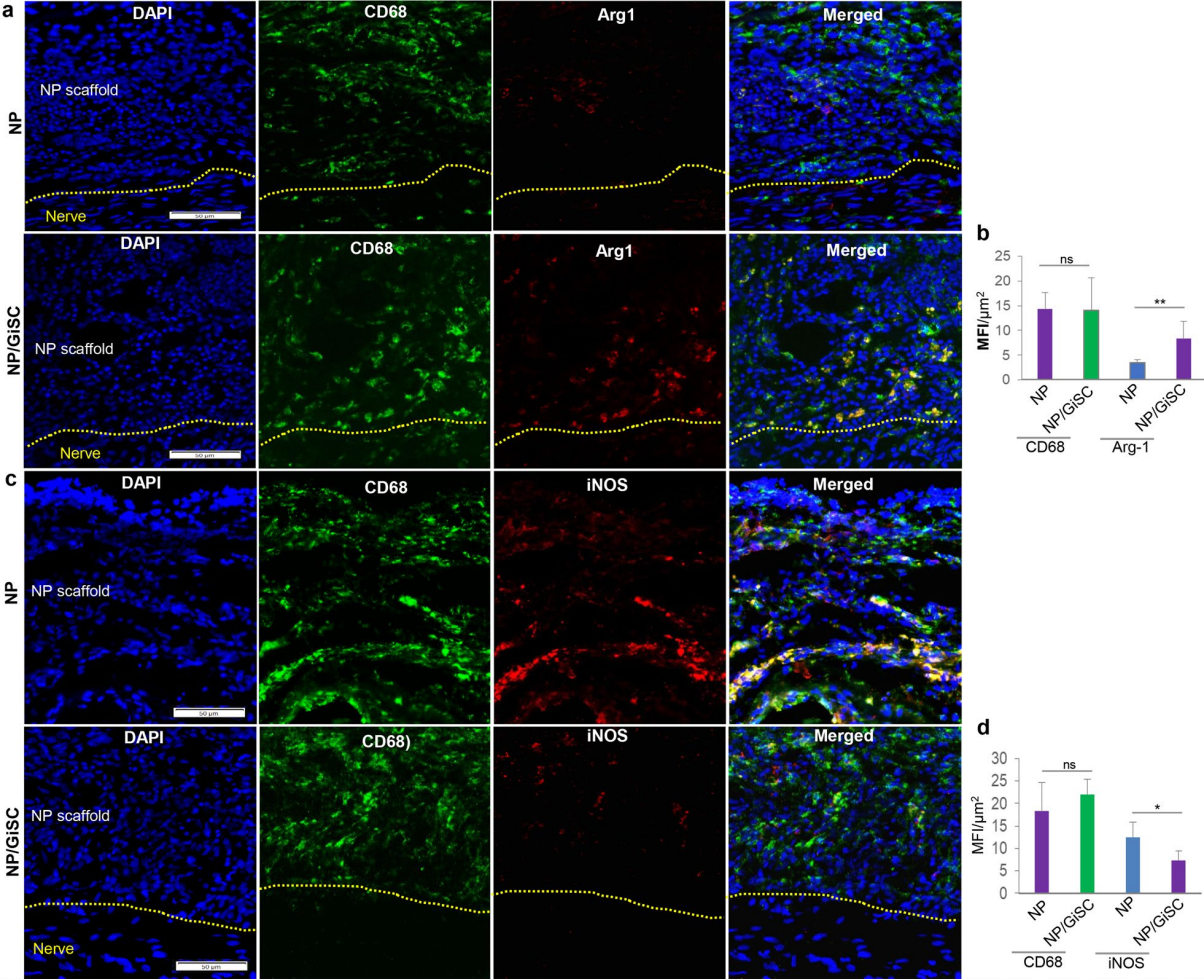
Immunomodulatory effects of GMSC-derived Schwann-like cells on pro-inflammatory (M1)/pro-regenerative (M2) macrophages in peripheral regions of crush-injured rat sciatic nerves. The functionalized nerve protectors repopulated with GMSC-derived Schwann-like cells were implanted to wrap the injured regions of rat sciatic nerves. Four weeks post-implantation, the injured nerves were harvested and cryosections were prepared for immunofluorescence studies. The cryosections were incubated with a specific mouse monoclonal antibody for rat CD68 (green color) in combination with a rabbit polyclonal antibody for arginase-1 (red color) followed by incubation with Alexa Fluor 488- and 594-conjugated secondary antibodies. Nuclei were counterstained with 4’,6-diamidino-2-phenylindole (DAPI; blue). Images were captured under a fluorescence microscope. Scale bars, 50 µm. The dashed lines separated the longitudinally sectioned nerve tissues (the lower side) and implanted nerve protector (NP) scaffolds (the upper side). **b** Semi-quantification of the integrated mean fluorescence intensity (MFI) for CD68 and arginase-1. Data are shown as the mean ± SD. ns, no significance; **p < 0.01; Student’s two-tailed unpaired *t* test. **c** The cryosections were incubated with a specific mouse monoclonal antibody for rat CD68 (green color) in combination with a rabbit polyclonal antibody for iNOS (red color) followed by incubation with Alexa Fluor 488- and 594-conjugated secondary antibodies. Nuclei were counterstained with (DAPI; blue). Images were captured under a fluorescence microscope. Scale bars, 50 µm. The dashed lines separated the longitudinally sectioned nerve tissues (the lower side) and implanted nerve protector (NP) scaffolds (the upper side). **d** Semi-quantification of the integrated mean fluorescence intensity (MFI) for CD68 and iNOS. Data are shown as the mean ± SD. ns, no significance; *p < 0.05; Student’s two-tailed unpaired t test. Abbreviations: NP, nerve protector; NP/GiSC, nerve protector repopulated with GMSC-derived Schwann-like cells (GiSC); Arg1, arginase-1; iNOS, inducible nitric oxide synthase

We showed that GMSC-derived Schwann-like cells (GiSCs) possess potent in vitro modulatory functions to promote the polarization of pro-regenerative (M2) macrophages, and concomitantly, inhibit the activation of pro-inflammatory (M1) macrophages, which are comparable to those conferred by their parental GMSC counterparts (Fig. 2). We then determined the effects of GiSCs on pro-inflammatory (M1)/pro-regenerative (M2) macrophages in the crush-injured sciatic nerves of rats. CD68 is a common marker for total macrophages, while arginase-1 (Arg-1) and inducible nitric oxide synthase (iNOS) are commonly used as signature genes for pro-regenerative (M2) and pro-inflammatory (M1) macrophages, respectively [7]. We then observed the infiltration of CD68^+^iNOS^+^ pro-inflammatory (M1) and CD68^+^Arg1^+^ pro-regenerative (M2) macrophages in the wall matrix of implanted NPs. Our results indicated that there was no obvious difference in the infiltration of total CD68^+^ macrophages in the wall matrix of empty NPs and that of GiSC-repopulated NPs at week 4 post-implantation (*p* > 0.05; Fig. 6b, d). However, there were a significant increase in the infiltration of CD68^+^Arg1^+^ pro-regenerative (M2) macrophages (*p* < 0.01; Fig. Figure 6a, b) but a decrease in the infiltration of CD68^+^iNOS^+^ pro-inflammatory (M1) macrophages (*p* < 0.05; Fig. 6c, d) in the wall matrix of GiSC-repopulated NPs compared to those in empty NPs. In addition, we also observed the infiltration of CD68^+^iNOS^+^ pro-inflammatory (M1) and CD68^+^Arg1^+^ pro-regenerative (M2) macrophages within the injured nerve tissues. Our results showed that there was no significant difference in the infiltration of total CD68^+^ macrophages within injured nerve controls compared with those wrapped with empty NPs at week 4 post-implantation (*p* > 0.05; Additional file 3: Fig. S3a, b; Additional file 4: Fig. S4a, b). On the contrary, the infiltration of total CD68^+^ macrophages was significantly reduced within injured nerves wrapped with GiSC-repopulated NPs compared to that in injured nerve controls or those wrapped with empty NPs (*p* < 0.001; Additional file 3: Fig. S3a, b; Additional file 4: Fig. S4a, b). Additionally, our results indicated that there was a relatively higher infiltration of CD68^+^Arg1^+^ pro-regenerative (M2) macrophages (*p* < 0.05; Additional file 3: Fig. S3a, b) but a lower infiltration of CD68^+^iNOS^+^ pro-inflammatory (M1) macrophages (*p* < 0.01; Additional file 4: Fig. S4a, b) within the injured nerves wrapped with GiSC-repopulated NPs compared to that in injured nerve controls or those wrapped with empty NPs. Taken together, these findings suggest that GMSC-derived Schwann-like cells retained potent capabilities to promote pro-regenerative (M2) macrophage polarization while suppressing pro-inflammatory (M1) macrophage activation in crush-injured sciatic nerves of rats.

## Discussion

PNIs remain one of the common challenges in clinical practice, among which traumatic compressive/crush injuries or axonotmesis are the most common type of lesions with the loss of integrity of the axon and myelin sheath while the perineural layer of connective tissue is usually intact [1–3]. Despite the advancement in microsurgical techniques and some other interventions, the degree of regeneration and functional recovery of severe crushed or transected injuries of peripheral nerves are usually unsatisfactory due to their restricted intrinsic or spontaneous regenerative potential [3]. Therefore, there is an increasing clinical demand for novel therapeutic approaches for the management of various PNIs.

In recent years, the combination of stem cell biology, biomaterial science, and tissue engineering technologies has shed light on developing different cell-based TE/RM products for efficacious therapy of PNIs [12, 13]. Due to the critical role in peripheral nerve repair/regeneration following injury, primary autologous or allogeneic Schwann cells may represent the most effective type of supportive cells to aid regeneration after they are transplanted to the nerve injury site [5, 8]. However, the major challenge facing the application of primary Schwann cells in peripheral nerve regeneration includes their limited availability and the difficulty in harvesting and ex vivo expansion of them [33]. Accumulating evidence has indicated that Schwann-like cells induced from different types of stem cells, including embryonic stem cells (ESC), induced pluripotent stem cells (iPSC), and various multipotent postnatal stem cells, represent important alternative sources of Schwann cells, among which mesenchymal stem/stromal cells (MSCs) readily isolated from a variety of tissues, such as bone marrow, skin, adipose, umbilical cord, and dental tissues, are the major source of Schwann-like cells for cell-based therapy of PNIs [33]. However, several limitations exist in differentiating MSCs into Schwann-like cells, such as the requirement of special induction medium, the time-consuming process (usually taking 2 ~ 3 weeks in special induction conditions) [48], the variation in the induction efficiency, and the phenotypic instability of MSC-derived Schwann-like cells, etc. [33, 49]. In recent years, various approaches have been developed to promote induction of MSCs into Schwann-like cells, particularly the use of certain small molecules or growth factors [48, 50] and engineered substrates or scaffolds such as graphene oxide substrate [51], 3D-printed polycaprolactone/polypyrrole (PCL/PPy) conductive scaffold [52], 3D-printed bionic polycaprolactone (PCL) polymer scaffold [53], and aligned nanofibers [54]. Consistent with our recent studies [37], we, herein, have also demonstrated that GMSCs could directly and rapidly convert into Schwann-like cells when they were encapsulated in methacrylated 3D-collagen hydrogel and cultured under the regular MSC culture medium without the additional introduction of special nutritional and growth factor supplements (Fig. 1). Therefore, it is worthy to explore whether GMSCs encapsulated in other type of scaffolds, e.g., fibrin, Matrigel, gelatin methacrylate (GelMA), and poly(ethyleneglycol) diacrylate (PEGDA) [55], etc., could also convert into Schwann-like cells. Additionally, it is noteworthy that our recent study indicated that BMSCs encapsulated in methacrylated 3D-collagen hydrogel and cultured under the same conditions as GMSCs failed to convert into Schwann-like cells [37]. Then, further studies are warranted to explore whether MSCs derived from other types of tissues, e.g., adipose, umbilical cord, and dental pulp, etc., could convert into Schwann-like cells when they were encapsulated in methacrylated 3D-collagen hydrogel and cultured under the same conditions as GMSCs.

Developmentally, Schwann cells and peripheral glial cells are derivatives of NCSCs and are essential for tissue homeostasis, regeneration, and disease pathogenesis [8, 56]. Recently, a unique subpopulation of MSCs endowed with NCSC-like properties has been isolated from various adult NC-derived tissues, such as dental pulp [15, 57], oral mucosa/gingiva [16–18, 35, 36], and skin epidermis [19, 58]. Compared to other sources of MSCs, these NC-derived MSCs appear to possess an intrinsic propensity to differentiate into glial or Schwann-like cells [19, 56, 59, 60]. Given that GMSCs are of a neural crest origin [18, 35, 36], our previous studies have shown that ex vivo expanded GMSCs could directly convert to NCSC-like or neural progenitor-like cells under defined culture conditions, which exerted better therapeutic effects on axonal regeneration and functional recovery of injured peripheral nerves than their parental GMSC counterparts [34, 41]. Recently, we demonstrated that differentiated GMSC-derived Schwann-like cells spontaneously self-assemble into aligned bundled cables in vitro, similar to the native bands of Büngner [61]. In agreement with our recent findings [37], our current study showed that GMSCs encapsulated in the methacrylated 3D-collagen hydrogel directly and rapidly converted into Schwann-like cells (GiSCs) which could migrate into the wall matrix of nerve protectors made from SIS-ECM, thus allowing the generation of functionalized nerve protectors. In addition, we found that among the infiltrated cells in the perineural areas that positively expressed the Schwann cell marker S-100β, GDNF, and BDNF, about 80% of them were exogenous human cells as presented by the expression of human nuclei (Fig. 3d). Those S-100β^+^, GDNF^+^, or BDNF^+^ cells but not co-expressing human nuclei might be host Schwann cells with an injury-induced repair phenotype characterized by enhanced proliferative, migratory, and invasive capabilities [5, 56], which might have migrated through the damaged epineural barrier to the perineural areas. Therefore, further studies are warranted to confirm the origin of these endogenous host cells. Importantly, we demonstrated that implantation of functionalized nerve protectors laden with GiSCs facilitated axonal regeneration and functional recovery in a sciatic nerve crush injury model in rats. Taken together, these studies suggest that adult NC-derived MSCs, particularly GMSCs, represent a promising source of Schwann-like cells for cell-based regenerative therapy of PNIs.

Upon severe peripheral nerve injuries, drastic disturbance in both physical barriers and physiological/immune homeostasis happens in the injured nerve microenvironment characterized by excessive inflammation, dysregulated metabolism, abnormal vascularization, and disrupted transduction of electric signals, etc., while such imbalanced neuronal microenvironment has dramatic negative effects on the regeneration and functional recovery of injured nerves [62]. Traditionally, nerve guidance conduits (NGCs) are designed to simply provide physical barriers and supportive guidance for axonal regrowth. Nevertheless, these commercially available NGCs only exhibit suboptimal beneficial effects on PNI with a small gap (< 30 mm) but little beneficial effects for PNI with a gap larger than 30 mm possibly because they are incapable of remodeling or reprogramming the disturbed neuronal microenvironment so that it can be favorable to nerve regeneration [9, 62, 63]. In recent years, various novel technologies or approaches have been incorporated to fabricate the new generation of NGCs composed of various types of biomimetic and functional scaffolds with modifications in their basic topological, biochemical, and physical properties, which not only provides physical neural support but can also rebalance the disturbed neuronal microenvironment through modulating the four key neural regeneration factors, including immune responses, intraneural vascularization, bioenergetic metabolism, and bioelectrical conduction [62, 63]. Such novel approaches include the design of different types of nanomaterials [64], scaffold surface modification [65], the development of grooved micro- and nanofibers [66] and boron nitride nanosheets (BNNS)-functionalized polycaprolactone (PCL) channel scaffold [67], and so on. For example, Qian Y et al. recently reported that the smart BNNS@PCL porous channel scaffold with high elasticity, hydrophilicity, and biocompatibility could stimulate cellular secretion of neurotrophic factors by increasing bioelectrical signal transduction under ultrasonic actuation in vitro [67]. Meanwhile, the BNNS@PCL scaffold could maintain Schwann cell viability by modulating reactive oxygen species (ROS) level and restoring the energy metabolic balance [67]. In vivo, they showed that implantation of the smart BNNS@PCL scaffold promotes neovascularization and significantly improves muscular atrophy in a sciatic nerve defect model [67]. Taken together, these studies have shed light on the development of the new generation of NGCs composed of biomimetic functionalized scaffolds that can remodel or rebalance the disturbed nerve injury-associated microenvironment so as to provide efficient regenerative therapy for PNI.

It is known that interactions between Schwann cells and immune cells, particularly macrophages, in the disturbed injury microenvironment play critical roles in the whole process of nerve repair/regeneration following injury [6, 7]. During the early phase of nerve injury, infiltrated macrophages are predominated by pro-inflammatory (M1) phenotypes and, subsequently, transit to an intermediate phenotype characterized by the co-expression of pro-inflammatory (M1) and pro-regenerative (M2) macrophage markers or predominant pro-regenerative (M2) phenotype [7]. Therefore, manipulation of the plastic phenotype of macrophages is emerging as a novel therapeutic approach for peripheral repair/regeneration [68]. In recent years, immunomodulatory or microenvironment-regulating biomaterials, either synthetic or naturally derived, have been fabricated to drive the polarization of macrophages toward an anti-inflammatory or pre-repair (M2) phenotype, thus facilitating tissue regeneration, including nerve repair [68]. For instance, a recent study showed that implantation of the electrospun nanofiber scaffolds promotes polarization of macrophages toward a pro-regenerative (M2) phenotype and a consequent decrease in the M1/M2 ratio and scarring/fibrosis at the nerve injury site, and concomitantly, a higher number of regenerated axons [69]. Most recently, Zhukauskas R et al. compared the subchronic rodent host responses to two commercially available nerve conduits, the Axoguard Nerve Connector, made of porcine small intestine submucosa (SIS) (Axogen), and the NeuraGen Nerve Guide, made of cross-linked bovine type I collagen (Col) (Integra Life Sci) at 4 weeks post-implantation. They found a significantly higher infiltration of M2 macrophages in response to the SIS Axoguard conduits but a predominant infiltration of M1 macrophages in response to the NeuraGen Nerve Guide conduits [70]. In addition to immunomodulatory biomaterials, it is well known that MSCs of different tissue origins can potently promote pro-regenerative (M2) macrophage polarization while inhibiting pro-inflammatory (M1) macrophage activation both in vitro and in various preclinical disease models [14]. MSCs exert their therapeutic effects on PNI possibly through their dual functions, multipotent differentiation capacity into neural type of cells and their paracrine secretion of an array of bioactive factors with multiple trophic effects on both neural and nonneural cells [13], whereby the potent immunomodulatory effects of MSCs on immune cells, particularly macrophages, may constitute one of the major mechanisms by which MSCs exert their therapeutic effects on PNI [14]. Our previous studies showed that GMSCs possess potent immunomodulatory effects on pro-inflammatory (M1)/pro-regenerative (M2) macrophages both in vitro and in mice skin wound models [39]. In the present study, we showed that GMSCs encapsulated in the 3D-collagen hydrogel converted into Schwann-like cells and retained the potent stimulatory effects on pro-regenerative (M2) macrophage polarization and inhibitory effects on pro-inflammatory (M1) macrophage activation in a co-culture system in vitro. Importantly, such effects of GMSC-derived Schwann-like cells on pro-inflammatory (M1)/pro-regenerative (M2) macrophages were recapitulated in crush-injured rat sciatic nerves following their implantation within SIS nerve protectors. According to previous studies, MSCs exert immunomodulatory effects on pro-inflammatory (M1)/pro-regenerative (M2) macrophages via the secretion of various soluble bioactive factors, such as IL-10, TGF-β, prostaglandin (PGE2), tumor-necrosis-factor-inducible gene 6 protein (TSG-6), and monocyte chemoattractant protein-1 (MCP-1).[14]. Further studies are warranted to elucidate the mechanisms by which GMSC-derived Schwann-like cells modulate pro-inflammatory (M1)/pro-regenerative (M2) macrophage polarization in the setting of PNI.

## Conclusions

In summary, Schwann-like cells converted from GMSCs retained potent immunomodulatory functions to promote pro-regenerative (M2) macrophage polarization and suppress pro-inflammatory (M1) macrophage activation. Implantation of functionalized nerve protectors repopulated GMSC-converted Schwann-like cells to accelerate axonal regeneration and functional recovery of crush-injured rat sciatic nerves accompanied by increased infiltration of pro-regenerative (M2) macrophages while a decreased infiltration of pro-inflammatory (M1) macrophages. These findings suggest that Schwann-like cells converted from GMSCs represent a promising source of supportive cells for regenerative therapy of PNI through their dual functions, neurotrophic effects, and immunomodulation of pro-inflammatory (M1)/pro-regenerative (M2) macrophages.

## Supplementary Information

**Additional file1: Fig. 1.** Survival ability of GMSC-derived Schwann-like cells following transplantation within the functionalized nerve proctor to the crush injury site of rat sciatic nerves. **a** The functionalized nerve protectors repopulated with GMSC-derived Schwann-like cells were implanted to wrap the injury segment of rat sciatic nerves. 4 weeks post-implantation, the injured nerves were harvested and cryosections were prepared for immunofluorescence studies. **b** The cryosections were incubated with a specific mouse monoclonal antibody for human nuclei (hNu; red color) in combination with a rabbit polyclonal antibody for the active form of caspase 3 (Casp-3) followed by incubation with Alexa Fluor 488- and 594-conjugated secondary antibodies. Nuclei were counterstained with 4’,6-diamidino-2-phenylindole (DAPI; blue). Yellow arrows indicate cells co-immunostaining with human nuclei (red) and Casp-3 (green) (Casp3+hNu+). **c** Quantification of the percentage of apoptosis in transplanted human MSCs presented by co-immunostaining with human nuclei (red color) and the active Casp-3 (green color) by using ImageJ. Images were captured under a fluorescence microscope. Scale bars, 50µm. The dashed lines separated the longitudinally sectioned nerve tissues (the lower side) and implanted neural protector (NP) scaffolds (the upper side).

**Additional file2: Fig. 2.** Implantation of nerve protectors repopulated with GMSC-derived Schwann-like cells promotes axonal regeneration of crush-injured rat sciatic nerves. The functionalized nerve protectors repopulated with GMSC-derived Schwann-like cells were implanted to wrap the injured regions of rat sciatic nerves. Four weeks post-implantation, the injured nerves were harvested and cryosections were prepared for immunofluorescence studies. **a** The cryosections were incubated with a specific mouse monoclonal antibody for β-tubulin III (red color) in combination with a rabbit polyclonal antibody for S-100β (green) followed by incubation with Alexa Fluor 488- and 594-conjugated secondary antibodies. Nuclei were counterstained with 4’,6-diamidino-2-phenylindole (DAPI; blue). Images were captured under a fluorescence microscope. Scale bars, 50µm. **b**, **c** Semi-quantification of the integrated mean fluorescence intensity (MFI) for S-100β and β-tubulin III. Data are shown as the mean ± SD. *p<0.05, **p<0.01, ***p<0.01. Student’s two-tailed unpaired t test. Abbreviations: NP, nerve protector; NP/GiSC, nerve protector repopulated with GMSC-derived Schwann-like cells (GiSC).

**Additional file3: Fig. 3.** Immunomodulatory effects of GMSC-derived Schwann-like cells on M2 macrophages within crush-injured rat sciatic nerves. The functionalized nerve protectors repopulated with GMSC-derived Schwann-like cells were implanted to wrap the injured regions of rat sciatic nerves. Four weeks post-implantation, the injured nerves were harvested and cryosections were prepared for immunofluorescence studies. **a** The cryosections were incubated with a specific mouse monoclonal antibody for rat CD68 (green color) in combination with a rabbit polyclonal antibody for arginase-1 (red color) followed by incubation with Alexa Fluor 488- and 594-conjugated secondary antibodies. Nuclei were counterstained with 4’,6-diamidino-2-phenylindole (DAPI; blue). Images were captured under a fluorescence microscope. Scale bars, 50µm. b Semi-quantification of the integrated mean fluorescence intensity (MFI) for CD68 and arginase-1. Data are shown as the mean ± SD. ns, no significance; *p<0.05; ***p<0.001. Student’s two-tailed unpaired t test. Abbreviations: NP, nerve protector; NP/GiSC, nerve protector repopulated with GMSC-derived Schwann-like cells (GiSC); Arg-1, arginase-1.

**Additional file4: Fig. 4.** Immunomodulatory effects of GMSC-derived Schwann-like cells on M1 macrophages within crush-injured rat sciatic nerves. The functionalized nerve protectors repopulated with GMSC-derived Schwann-like cells were implanted to wrap the injured regions of rat sciatic nerves. Four weeks post-implantation, the injured nerves were harvested and cryosections were prepared for immunofluorescence studies. **a** The cryosections were incubated with a specific mouse monoclonal antibody for rat CD68 (green color) in combination with a rabbit polyclonal antibody for iNOS (red color) followed by incubation with Alexa Fluor 488- and 594-conjugated secondary antibodies. Nuclei were counterstained with 4’,6-diamidino-2-phenylindole (DAPI; blue). Images were captured under a fluorescence microscope. Scale bars, 50µm. **b** Semi-quantification of the integrated mean fluorescence intensity (MFI) for CD68 and iNOS. Data are shown as the mean ± SD. ns, no significance; *p<0.05; ***p<0.001. Student’s two-tailed unpaired *t* test. Abbreviations: NP, nerve protector; NP/GiSC, nerve protector repopulated with GMSC-derived Schwann-like cells (GiSC); iNOS, inducible nitric oxide synthase.

## Abbreviations

PNIs: Peripheral nerve injuries
MSCs: Mesenchymal stem cells
GMSCs: Gingiva-derived mesenchymal stem cells
GiSC: GMSC-derived Schwann-like cells
α-MEM: α-Minimum essential medium
FBS: Fetal bovine serum
NEAA: Non-essential amino acid
2-ME: 2-Mercaptoethanol
IL-10: Interleukin-10
TNF-α: Tumor necrosis factor-α
IL-1β: Interleukin-1β
ELISA: Enzyme-linked immunoassay
SIS-ECM: Small intestine submucosal extracellular matrix
BDNF: Brain-derived neurotrophic factor
GDNF: Glial cell-derived neurotrophic factor
LPS: Lipopolysaccharide
NC: Neural crest
NCSC: Neural crest-derived stem cell
TE/RM: Tissue engineering/regenerative medicine
