# Supplementary material for: Implantation of a nerve protector embedded with human GMSC-derived Schwann-like cells accelerates regeneration of crush-injured rat sciatic nerves
Source: Stem Cell Res Ther. 2022 Jun 20;13:263. doi: 10.1186/s13287-022-02947-4 (PMC9208168; doi:10.1186/s13287-022-02947-4)
Supplement: Supplementary file 1 — Additional file1: Fig. 1. Survival ability of GMSC-derived Schwann-like cells following transplantation within the functionalized nerve proctor to the crush injury site of rat sciatic nerves. a The functionalized nerve protectors repopulated with GMSC-derived Schwann-like cells were implanted to wrap the injury segment of rat sciatic nerves. 4 weeks post-implantation, the injured nerves were harvested and cryosections were prepared for immunofluorescence studies. b The cryosections were incubated with a specific mouse monoclonal antibody for human nuclei (hNu; red color) in combination with a rabbit polyclonal antibody for the active form of caspase 3 (Casp-3) followed by incubation with Alexa Fluor 488- and 594-conjugated secondary antibodies. Nuclei were counterstained with 4’,6-diamidino-2-phenylindole (DAPI; blue). Yellow arrows indicate cells co-immunostaining with human nuclei (red) and Casp-3 (green) (Casp3+hNu+). c Quantification of the percentage of apoptosis in transplanted human MSCs presented by co-immunostaining with human nuclei (red color) and the active Casp-3 (green color) by using ImageJ. Images were captured under a fluorescence microscope. Scale bars, 50µm. The dashed lines separated the longitudinally sectioned nerve tissues (the lower side) and implanted neural protector (NP) scaffolds (the upper side). [file 13287_2022_2947_MOESM1_ESM.doc]

**
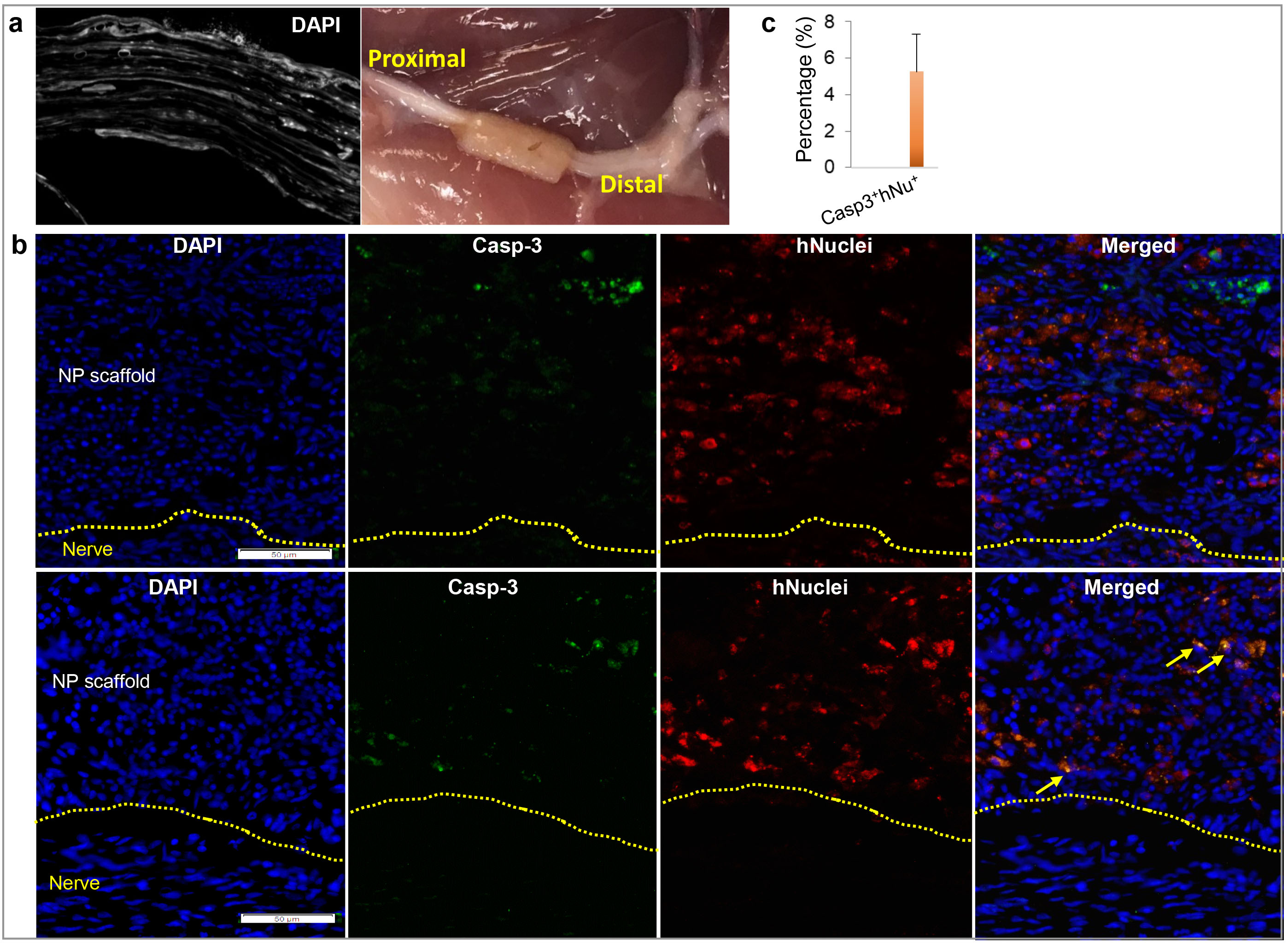
**

**Supplemental Fig. 1** Survival ability of GMSC-derived Schwann-like cells following transplantation within the functionalized nerve proctor to the crush injury site of rat sciatic nerves. **a** The functionalized nerve protectors repopulated with GMSC-derived Schwann-like cells were implanted to wrap the injury segment of rat sciatic nerves. 4 weeks post-implantation, the injured nerves were harvested and cryosections were prepared for immunofluorescence studies. **b** The cryosections were incubated with a specific mouse monoclonal antibody for human nuclei (hNu; red color) in combination with a rabbit polyclonal antibody for the active form of caspase 3 (Casp-3) followed by incubation with Alexa Fluor 488- and 594-conjugated secondary antibodies. Nuclei were counterstained with 4’, 6-diamidino-2-phenylindole (DAPI; blue). Yellow arrows indicate cells co-immunostaining with human nuclei (red) and Casp-3 (green) (Casp3^+^hNu^+^). **c** Quantification of the percentage of apoptosis in transplanted human MSCs presented by co-immunostaining with human nuclei (red color) and the active Casp-3 (green color) by using ImageJ. Images were captured under a fluorescence microscope. Scale bars, 50µm. The dashed lines separated the longitudinally sectioned nerve tissues (the lower side) and implanted neurol protector (NP) scaffolds (the upper side).
