# Supplementary material for: Implantation of a nerve protector embedded with human GMSC-derived Schwann-like cells accelerates regeneration of crush-injured rat sciatic nerves
Source: Stem Cell Res Ther. 2022 Jun 20;13:263. doi: 10.1186/s13287-022-02947-4 (PMC9208168; doi:10.1186/s13287-022-02947-4)
Supplement: Supplementary file 2 — Additional file2: Fig. 2. Implantation of nerve protectors repopulated with GMSC-derived Schwann-like cells promotes axonal regeneration of crush-injured rat sciatic nerves. The functionalized nerve protectors repopulated with GMSC-derived Schwann-like cells were implanted to wrap the injured regions of rat sciatic nerves. Four weeks post-implantation, the injured nerves were harvested and cryosections were prepared for immunofluorescence studies. a The cryosections were incubated with a specific mouse monoclonal antibody for β-tubulin III (red color) in combination with a rabbit polyclonal antibody for S-100β (green) followed by incubation with Alexa Fluor 488- and 594-conjugated secondary antibodies. Nuclei were counterstained with 4’,6-diamidino-2-phenylindole (DAPI; blue). Images were captured under a fluorescence microscope. Scale bars, 50µm. b, c Semi-quantification of the integrated mean fluorescence intensity (MFI) for S-100β and β-tubulin III. Data are shown as the mean ± SD. *p<0.05, **p<0.01, ***p<0.01. Student’s two-tailed unpaired t test. Abbreviations: NP, nerve protector; NP/GiSC, nerve protector repopulated with GMSC-derived Schwann-like cells (GiSC). [file 13287_2022_2947_MOESM2_ESM.doc]

**
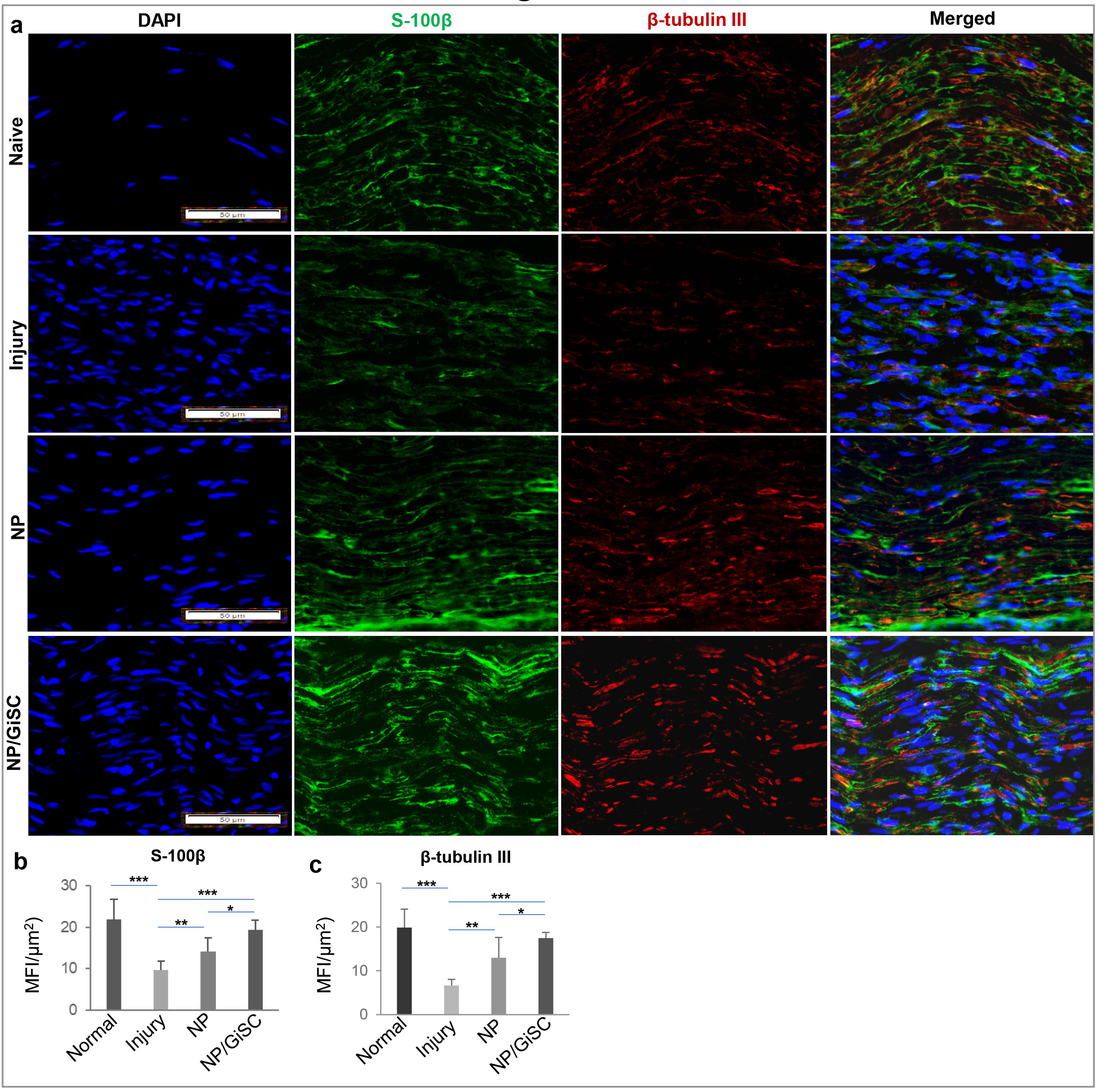
**

**Supplemental Fig. 2** Implantation of nerve protectors repopulated with GMSC-derived Schwann-like cells promotes axonal regeneration of crush-injured rat sciatic nerves. The functionalized nerve protectors repopulated with GMSC-derived Schwann-like cells were implanted to wrap the injured regions of rat sciatic nerves. 4 weeks post-implantation, the injured nerves were harvested and cryosections were prepared for immunofluorescence studies. **a** The cryosections were incubated with a specific mouse monoclonal antibody for β-tubulin III (red color) in combination with a rabbit polyclonal antibody for S-100β (green) followed by incubation with Alexa Fluor 488- and 594-conjugated secondary antibodies. Nuclei were counterstained with 4’, 6-diamidino-2-phenylindole (DAPI; blue). Images were captured under a fluorescence microscope. Scale bars, 50µm. **b**, **c** Semi-quantification of the integrated mean fluorescence intensity (MFI) for S-100β and β-tubulin III. Data are shown as the mean ± SD. **p*<0.05, ***p*<0.01, ****p*<0.01. Student’s two-tailed unpaired t-test. Abbreviations: NP, nerve protector; NP/GiSC, nerve protector repopulated with GMSC-derived Schwann-like cells (GiSC).
