# Supplementary material for: Implantation of a nerve protector embedded with human GMSC-derived Schwann-like cells accelerates regeneration of crush-injured rat sciatic nerves
Source: Stem Cell Res Ther. 2022 Jun 20;13:263. doi: 10.1186/s13287-022-02947-4 (PMC9208168; doi:10.1186/s13287-022-02947-4)
Supplement: Supplementary file 4 — Additional file4: Fig. 4. Immunomodulatory effects of GMSC-derived Schwann-like cells on M1 macrophages within crush-injured rat sciatic nerves. The functionalized nerve protectors repopulated with GMSC-derived Schwann-like cells were implanted to wrap the injured regions of rat sciatic nerves. Four weeks post-implantation, the injured nerves were harvested and cryosections were prepared for immunofluorescence studies. a The cryosections were incubated with a specific mouse monoclonal antibody for rat CD68 (green color) in combination with a rabbit polyclonal antibody for iNOS (red color) followed by incubation with Alexa Fluor 488- and 594-conjugated secondary antibodies. Nuclei were counterstained with 4’,6-diamidino-2-phenylindole (DAPI; blue). Images were captured under a fluorescence microscope. Scale bars, 50µm. b Semi-quantification of the integrated mean fluorescence intensity (MFI) for CD68 and iNOS. Data are shown as the mean ± SD. ns, no significance; *p<0.05; ***p<0.001. Student’s two-tailed unpaired t test. Abbreviations: NP, nerve protector; NP/GiSC, nerve protector repopulated with GMSC-derived Schwann-like cells (GiSC); iNOS, inducible nitric oxide synthase. [file 13287_2022_2947_MOESM4_ESM.doc]

**
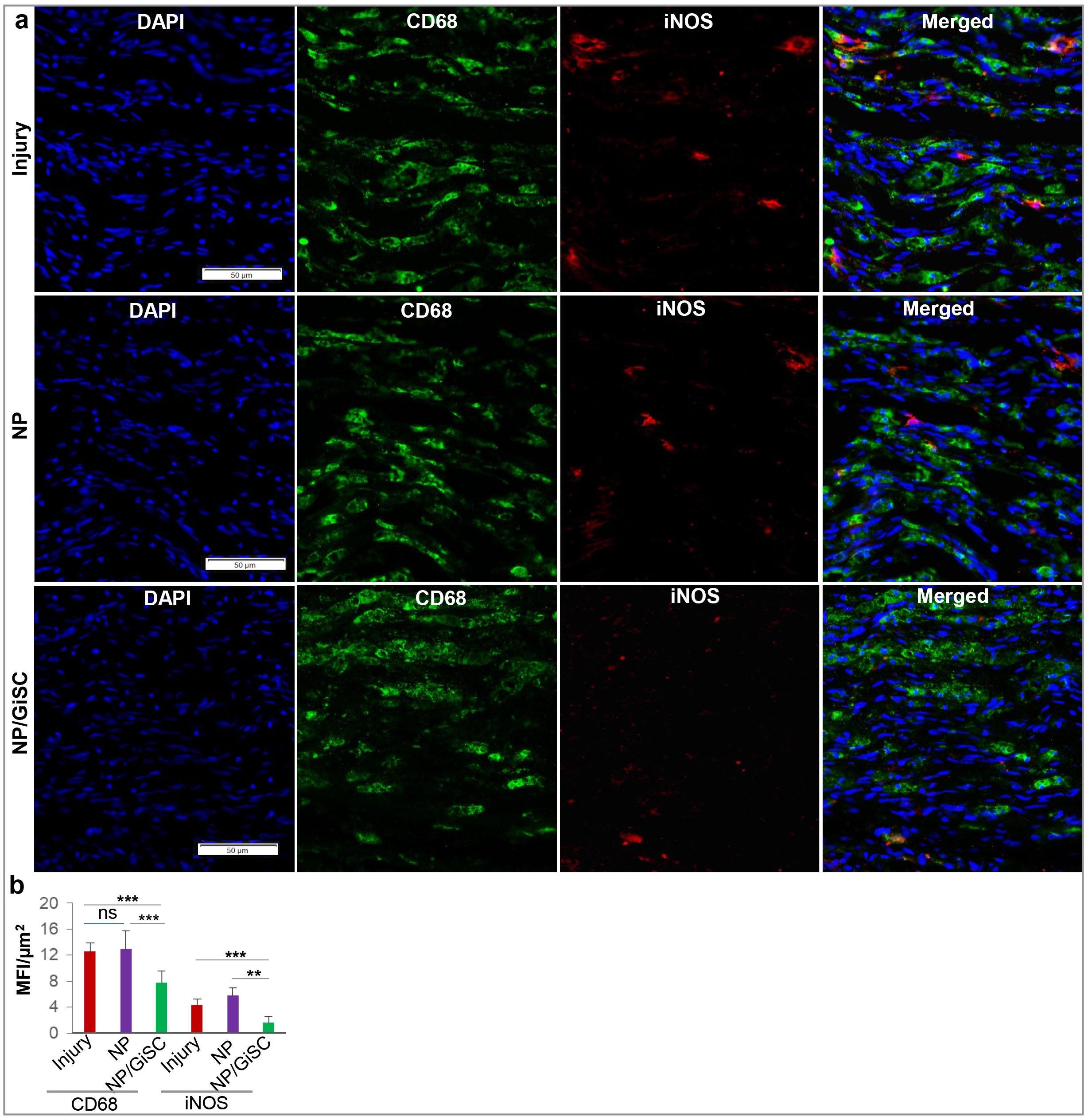
**

**Supplemental Fig. 4** Immunomodulatory effects of GMSC-derived Schwann-like cells on M1 macrophages within crush-injured rat sciatic nerves. The functionalized nerve protectors repopulated with GMSC-derived Schwann-like cells were implanted to wrap the injured regions of rat sciatic nerves. 4 weeks post-implantation, the injured nerves were harvested and cryosections were prepared for immunofluorescence studies. **a** The cryosections were incubated with a specific mouse monoclonal antibody for rat CD68 (green color) in combination with a rabbit polyclonal antibody for iNOS (red color) followed by incubation with Alexa Fluor 488- and 594-conjugated secondary antibodies. Nuclei were counterstained with 4’, 6-diamidino-2-phenylindole (DAPI; blue). Images were captured under a fluorescence microscope. Scale bars, 50µm. **b** Semi-quantification of the integrated mean fluorescence intensity (MFI) for CD68 and iNOS. Data are shown as the mean ± SD. ns, no significance; **p*<0.05; ****p*<0.001. Student’s two-tailed unpaired t-test. Abbreviations: NP, nerve protector; NP/GiSC, nerve protector repopulated with GMSC-derived Schwann-like cells (GiSC); iNOS, inducible nitric oxide synthase.
